# A 1-Tetradecanol-1, 10-Decanediol Binary Eutectic Mixture/Expanded Graphite Composite Phase Change Materials for Thermal Energy Storage

**DOI:** 10.3390/ma19020371

**Published:** 2026-01-16

**Authors:** Jun Yi, Rongjun Hu, Gaofei Zhan, Qiu Zeng, Jiyong Zou, Yu Xie, Shengyong You

**Affiliations:** 1 School of Environment and Chemical Engineering, Nanchang Hangkong University, Nanchang 330063, China; 2 Institute of Materials and Intelligent Manufacturing, Jiangxi Academy of Sciences, Nanchang 330096, China; 3 School of Chemistry and Materials Science, East China University of Technology, Nanchang 330013, China

**Keywords:** phase change material, 1-tetradecanol, 1,10-decanediol, expanded graphite

## Abstract

Organic phase change materials show potential for thermal energy storage, but their scalable implementation is limited by fixed phase change temperatures, molten leakage, and low thermal conductivity. To address the temperature constraint, a binary eutectic system of 1-tetradecanol and 1,10-decanediol is prepared, expanding the operational temperature range for building thermal management. Compositing the eutectic with expanded graphite yields a composite material that exhibits a low leakage and a markedly improved thermal conductivity of 4.642 W/(m·K), which is approximately 12 times that of the pure eutectic. The composite maintains distinct phase transition properties, with melting and solidification temperatures of 37.77 °C and 29.38 °C and corresponding latent heats of 218.80 J/g and 216.66 J/g. It also demonstrates a good cycling stability, retaining over 87% of the original latent heat after 2000 thermal cycles. While these findings remain valid under controlled conditions, further studies are required to evaluate their practical feasibility and long-term durability in real-world scenarios. This work establishes a systematic approach for fabricating composite phase change materials and provides a promising candidate for building thermal management applications.

## 1. Introduction

In the context of the global energy structure’s accelerating transition towards a low-carbon model, efficient energy storage technologies have become a crucial approach to alleviating the contradiction between energy supply and demand and enhancing the utilization efficiency of renewable energy [1,2,3]. The thermal energy storage (TES) system serves as a key component in energy management, facilitating energy balance and enhanced system efficiency [4,5,6]. Phase change materials (PCMs) are considered attractive candidates for TES systems owing to their high energy storage density, stable phase change temperature, and good reversibility [7,8]. Organic PCMs, particularly fatty alcohols, fatty acids, and related derivatives, offer a compelling combination of properties for TES, including a good chemical stability, limited supercooling, safe operation, and cost-effectiveness [9,10,11].

Nevertheless, the practical application of single-component organic phase change materials (PCMs) is hindered by several major limitations. Firstly, their fixed phase change temperature limits their adaptability to diverse application scenarios [12]. Secondly, the inherently low thermal conductivity constrains the thermal response rate, thus limiting the overall efficiency of TES and release [13]. To address these challenges, recent studies have increasingly explored multicomponent composite phase change systems. The constructions of binary or multicomponent eutectic mixtures enable the effective regulation of phase change temperature and improved thermal properties through synergistic effects. This approach offers alternative pathways for developing advanced TES materials [14,15,16].

1-Tetradecanol (TD) represents a typical organic PCM with a high latent heat (approximately 245 J/g) and a suitable phase change temperature (approximately 39 °C), showing promising applications in building energy efficiency, electronic device thermal management, and solar energy utilization [17]. However, the fixed phase change temperature of TD limits its adaptability over wider temperature ranges. Previous studies have attempted to modulate the thermal properties of TD through composite formation. For instance, Tang et al. [18] developed an octanoic acid-1-tetradecanol/expanded graphite-hexadecyltrimethoxysilane (OA-TD/EG-HDTMOS) composite with a phase change temperature approximately 10 °C lower than pure TD for building air-conditioning systems, while Cheng et al. [19] fabricated a 1-tetradecanol-palmitic acid/expanded perlite (TD-PA/EP-CF) composite showing a 5 °C reduction, extending its applicability to building envelopes and solar thermal storage.

1,10-Decanediol (DD), as another fatty alcohol-based PCM, exhibits a high latent heat (approximately 242 J/g) and a phase change temperature around 73 °C, which makes it suitable for medium-temperature industrial waste heat recovery [20]. However, the high phase change temperature of DD limits its direct application in building energy conservation. In contrast, combining TD with DD produces a binary eutectic system that allows the phase change temperature to be tuned within the range suitable for building applications. This approach also facilitates the effective utilization of the substantial latent heat from both components, leading to an improved thermal performance and overcoming the limitations found in earlier composite systems.

Although binary eutectic systems have solved the problem of adjustable phase change temperature to some extent, organic PCMs still face core scientific issues such as liquid phase leakage and low thermal conductivity. To address the leakage issue, methods such as porous matrix adsorption are commonly employed [21,22]. Expanded graphite (EG) is commonly employed as a supporting matrix for PCMs owing to its porous structure, good adsorption capability, and high thermal conductivity. Vacuum impregnation enables the integration of eutectic PCMs within the three-dimensional network of EG. This process improves shape stability and increases thermal conductivity, thus enabling a substantial energy storage density and effective heat transfer [23,24].

**Figure 1:**
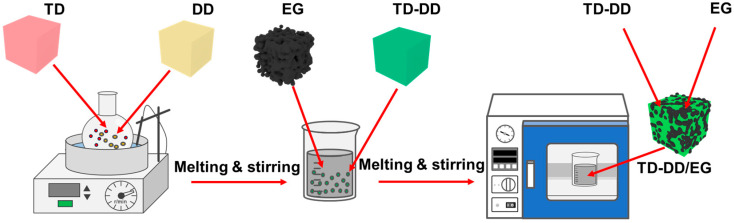
Schematic illustration of the preparation procedure for the TD-DD/EG composite PCM.

This study develops a TD-DD/EG composite PCM to address several common limitations of organic PCMs, including constrained temperature regulation, leakage tendency, and low thermal conductivity. As shown in Figure 1, the composite is prepared through vacuum impregnation. An optimal TD-DD eutectic mass ratio of 8:1 is identified by combining theoretical prediction with experimental validation. The resulting eutectic mixture exhibits melting and solidification temperatures of 37.89 °C and 29.90 °C, with latent heats of 226.00 J/g and 216.00 J/g, respectively. The TD-DD/EG composite maintains similar phase transition characteristics, showing melting at 37.77 °C (218.80 J/g) and solidification at 29.38 °C (216.66 J/g). The composite demonstrates minimal leakage after 2 h at 60 °C and achieves a thermal conductivity of 4.642 W/(m·K), which is 12 times that of the pure TD-DD eutectic. After 1000 thermal cycles, the material retains over 93.00% of its original latent heat. Further extended cycling to 2000 cycles reveals that it still maintains approximately 87.60% of the initial latent heat, with a decelerating degradation rate, demonstrating a good long-term cycling stability. While these results are promising, further investigations remain necessary to evaluate the behavior of the material under realistic application environments and extended service conditions. This work establishes a systematic framework for developing composite PCMs and provides relevant insights for building energy efficiency applications.

## 2. Materials and Methods

### 2.1. Experimental Materials and Instruments

1-Tetradecanol (TD, 98% pure) and 1, 10-Decanediol (DD, 98% pure) were obtained from Shanghai Titan Scientific Co.,Ltd, Shanghai, China. Expanded graphite (EG, 80 meshes) was supplied by Su Qian Graphene Valley Nano Technology Co., Ltd, SuQian, China.

The composite PCMs were systematically characterized using multiple techniques. Structural and chemical characterization included Fourier Transform Infrared (FT-IR) Spectroscopy (Nicolet iS5, Thermo Scientific, Waltham, MA, USA) with KBr pellet samples scanned over 400–4000 cm^−1^ at 4 cm^−1^ resolution to identify functional groups and chemical interactions. Crystallographic analysis was performed with X-ray Diffractometry (XRD) (XRD-7000, Shimadzu, Shanghai, China) using Cu Kα radiation (λ = 1.5406 Å) at 40 kV and 30 mA, with scans from 5° to 90° at 2°/min.

Thermal behavior was evaluated using Differential Scanning Calorimetry (DSC) (DSC7020, Tokyo, Japan). Samples of 4–6 mg were sealed in nitrogen-purged aluminum crucibles and subjected to thermal cycling between 10 °C and 60 °C at 5 °C/min under nitrogen atmosphere. Morphological characterization was conducted via Scanning Electron Microscopy (SEM) (Merlin, Zeiss, Jena, Germany) at a 5 kV accelerating voltage. Porous structure parameters were determined through nitrogen physisorption at 77 K (ASAP 2460, Micromeritics, Norcross, GA, USA), with the specific surface area calculated using the Brunauer–Emmett–Teller (BET) method and pore size distribution derived from the Barrett–Joyner–Halenda (BJH) model.

The thermal performance assessment included three key aspects: Thermal conductivity was measured using a Hot Disk TPS 2500S (Gothenburg, Sweden) analyzer with the transient plane source method. A Kapton-insulated sensor (radius 3.189 mm) was sandwiched between two identical cylindrical samples (4 mm thickness) under constant pressure applied by a 3 kg copper block, with all measurements conducted at 25 °C in triplicate. Thermal stability was evaluated through Thermogravimetric (TG) Analysis (STA 2500, NETZSCH, Selb, Germany), heating 3–10 mg samples from 30 °C to 600 °C at 10 °C/min under nitrogen. Cyclability testing (GM-05, Haoxing Biotechnology, Xi’an, China) was performed using a climate chamber with repeated melting–freezing cycles between 10 °C and 60 °C.

### 2.2. Preparation of Composite PCM

#### 2.2.1. Preparation of TD-DD Composite PCM

The optimal eutectic composition was identified by integrating theoretical predictions with experimental verifications. Based on theoretical calculations using the Schrader equation, which predicted an optimal mass ratio near 8:1, a series of TD-DD composite materials with systematically varied mass ratios were prepared for experimental screening. Specifically, for each formulation, the components were accurately weighed according to the designed ratio and heated to 90 °C under continuous stirring for 2 h until fully melted and homogenized. After cooling to room temperature, the solidified products were ground into fine powders to obtain the corresponding TD-DD composite PCMs for subsequent characterization. This combined approach ultimately confirmed the 8:1 ratio as the optimal eutectic composition.

#### 2.2.2. Preparation of TD-DD/EG Composite PCM

EG was dehydrated for 1 h at 105 °C in a constant-pressure drying oven (Model: 101-1AB, Tianjin Tester Instrument Co., Ltd., Tianjin, China) to eliminate physically adsorbed moisture. The desiccated EG was then combined with the optimized TD-DD eutectic mixture at systematically varied mass ratios (for example, 1.0000 g of TD-DD with 0.1000 g of EG for a 10:1 ratio) spanning from 10:1 to 17:1 (specifically, 10:1, 11:1, 12:1, 13:1, 14:1, 15:1, 16:1, and 17:1) to identify the optimal composition for shape stabilization. For each ratio, the corresponding precise quantities of TD-DD and EG were weighed accordingly. The mixtures were sealed in vessels, vacuum-packaged, and transferred to a temperature-programmable drying chamber. Under controlled vacuum conditions (−0.1 MPa), the systems underwent ambient-temperature impregnation for 4 h, followed by a thermal activation phase at 45 °C. During this stage, systematic mechanical agitation was applied at intervals of 30 min to ensure homogeneous dispersion within the EG matrix over a reaction period of 2 h. After synthesis, the systems were cooled to ambient conditions, yielding the final TD-DD/EG composite PCM for subsequent leakage tests and performance evaluations.

## 3. Results

### 3.1. Determination of TD:DD Ratio

Within eutectic theory, a eutectic mixture is characterized by its homogeneous nature and simultaneous phase transition at a distinct eutectic temperature, possessing the lowest melting point in the system with a satisfactory operational stability. Based on this theory, the optimal eutectic ratio for the binary organic PCM system is determined using the Schroder equation, as formulated in Equation (1) [25,26]:

(1) $$T_{m}=\frac{T_{i}H_{i}}{H_{i}-T_{i}RLnX_{i}}$$

where *T_m_* is the melting point of the mixture (°C); *T_i_* represents the melting points of components *A* and *B* (°C); R is the gas constant, R = 8.314 J/(mol·K); *X_i_* denotes the mole fractions of *A* and *B*, satisfying *X_A_ + X_B_* = 1; *H_i_* refers to the enthalpies of fusion of *A* and *B* (J/mol); and *A* and *B* represent the two components. Substituting the normalized melting temperatures and enthalpies of TD and DD into Equation (1) yields the following expression, designated as Equation (2):

(2) $$LnX_{TD}=1.85+1.02LnX_{DD}$$

Theoretical calculations predict a molar ratio of 6.38:1 for the TD-DD system, which corresponds to an approximate mass ratio of 8:1.

To validate this prediction, a series of composites with mass ratios near this value are prepared and evaluated. The experimental results confirm that the 8:1 formulation exhibits the best thermal performance among the prepared formulations, with a latent heat capacity of 226.00 J/g and a phase transition temperature of 37.89 °C. Accordingly, this composition is selected as the optimal candidate for further investigation.

### 3.2. Ratio Determination of EG to TD-DD via Leakage Test

**Figure 2:**
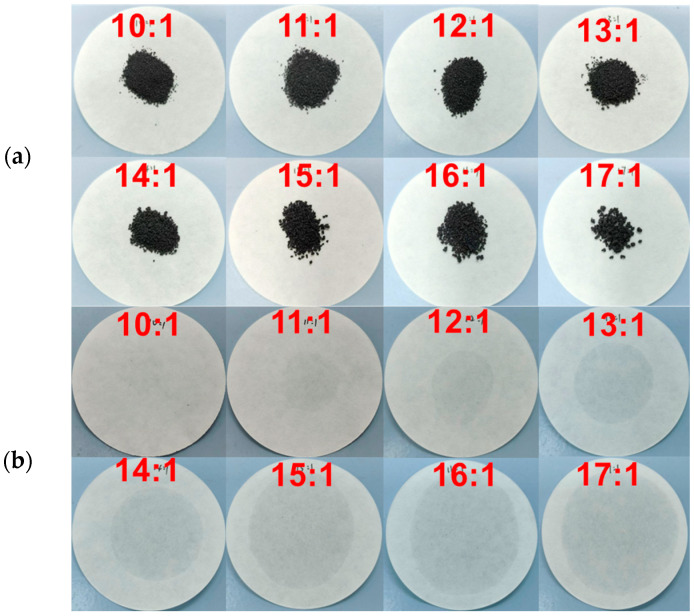
Digital photographs of the TD-DD/EG composite PCM (**a**) before and (**b**) after heat treatment at 60 °C for 2 h.

**Figure 3:**
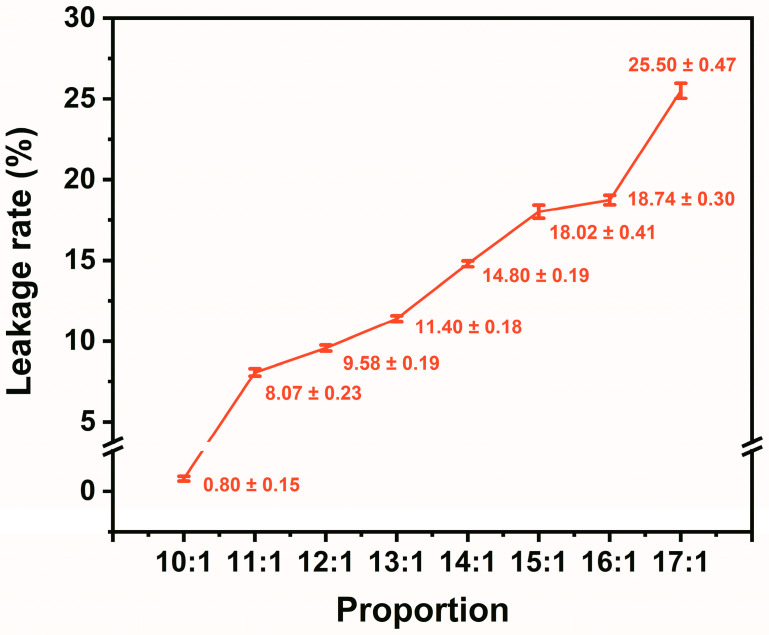
Leakage rates of TD-DD/EG composites with different mass ratios after heat treatment at 60 °C for 2 h.

Due to the capillary action and surface tension of EG, TD-DD is efficiently adsorbed into the porous network, effectively preventing liquid leakage during phase transition [27]. Leakage tests are conducted to determine the optimal EG content, ensuring a maximum energy storage capacity and shape stability. In this procedure, 0.2000 g of TD-DD/EG composite PCM with different mass ratios is placed on filter paper at the designated positions, as illustrated in Figure 2a, and then heated in an oven at 60 °C for 2 h. The results are shown in Figure 2b. A comparison of the PCM before and after heat treatment shows that leakage becomes apparent beyond the 10:1 ratio. The leakage rate is calculated using Equation (3):

(3) $$Leakage\;rate\left(100\%\right)=\frac{M_{a}-M_{b}}{M_{a}}\times 100\%$$

where *M_a_* is the mass of TD-DD/EG before heat treatment and *M_b_* is the mass of TD-DD/EG after heat treatment. As illustrated in Figure 3, the composite PCM with a 10:1 mass ratio exhibits a low leakage rate of 0.80%. The small standard deviation at this optimal ratio indicates a reproducible leakage prevention performance and confirms the uniform encapsulation of TD-DD within the EG matrix.

### 3.3. Crystal and Chemical Structure

**Figure 4:**
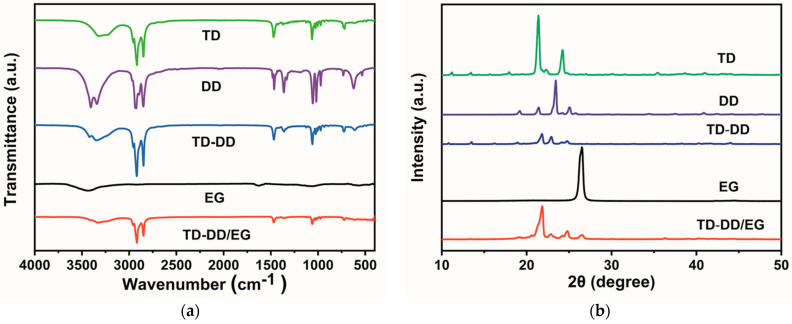
(**a**) FT-IR spectra and (**b**) XRD patterns of TD, DD, TD-DD, and the TD-DD/EG composite.

Figure 4a displays the FT-IR spectra of TD, DD, TD-DD, EG, and the TD-DD/EG composite PCM. In the TD-DD spectrum, the characteristic peaks at 2920 cm^−1^ and 2848 cm^−1^ are attributed to the symmetric and asymmetric stretching vibrations of the –CH_3_ and –CH_2_– groups in its components TD and DD, respectively, while the absorption peak at 1467 cm^−1^ corresponds to the bending vibration of C–H bonds. The out-of-plane bending vibrations of C–C bonds appear near 1060 cm^−1^ and 726 cm^−1^. The absorption band around 1060 cm^−1^ can be further assigned to the combined contributions of the in-plane bending vibration of C atoms in TD and the stretching vibration of C–O bonds in DD. In the spectrum of EG, characteristic absorptions corresponding to C=O, C–O, and O–O functional groups are observed. All spectra exhibit a broad absorption band above 3000 cm^−1^, indicating the presence of O–H groups [28]. Notably, the spectrum of the TD-DD/EG composite only shows the characteristic peaks of the individual components without the emergence of new peaks, confirming that the combination of EG with TD-DD occurs through physical interactions without chemical reactions.

The crystal structures of the PCMs are characterized through XRD, as shown in Figure 4b. EG exhibits a strong diffraction peak at 26.03°, corresponding to the (002) crystal plane with an interlayer spacing of 3.420 Å. TD displays two characteristic diffraction peaks at 21.77° and 24.85°, corresponding to interplanar spacings of 4.079 Å and 3.580 Å, respectively, while DD shows its main diffraction peak at 22.85°, with an interplanar spacing of 3.889 Å. Notably, the XRD pattern of TD-DD contains all the characteristic peaks of both TD and DD without noticeable peak shifting. In the XRD pattern of the TD-DD/EG composite, the characteristic peaks of EG as well as the complete diffraction features of TD and DD are well preserved. Apart from some variations in relative peak intensities, typically attributed to differences in crystal morphology, no new diffraction peaks are observed [29]. These results collectively demonstrate that TD-DD is formed through the physical blending of TD and DD, and its combination with EG likewise constitutes a physical process.

### 3.4. Pore Structure Analysis

**Figure 5:**
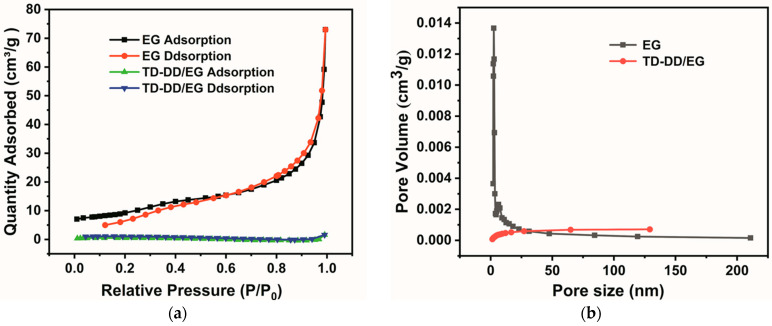
(**a**) Nitrogen adsorption–desorption curves and (**b**) pore size distribution curves for EG and TD-DD/EG.

The porous structure of the eutectic composite PCM is analyzed using BET measurements. The resultant nitrogen adsorption–desorption isotherms of EG and TD-DD/EG are presented in Figure 5, with corresponding structural parameters provided in Table 1. For EG, nitrogen adsorption commences at relative pressure (P/P_0_) values above 0.01. A pronounced hysteresis loop appears when P/P_0_ exceeds 0.50, confirming the coexistence of micropores and mesopores within the EG matrix [30,31]. Pristine EG exhibits a high specific surface area of 41.22 m^2^/g and a total pore volume of 0.1116 cm^3^/g, characteristics that provide a substantial capacity and interfacial area for the adsorption and encapsulation of the TD-DD eutectic mixture. In striking contrast, after vacuum impregnation, the TD-DD/EG composite displays markedly reduced values, with the specific surface area plummeting to 0.12 m^2^/g and the pore volume decreasing to 0.0010 cm^3^/g. This pronounced reduction in both surface area and pore volume provides strong evidence that the porous network of EG is almost entirely occupied by the TD-DD PCM, confirming a successful and nearly complete pore filling and the effective formation of a shape-stabilized composite.

### 3.5. Microstructure and Morphology

**Figure 6:**
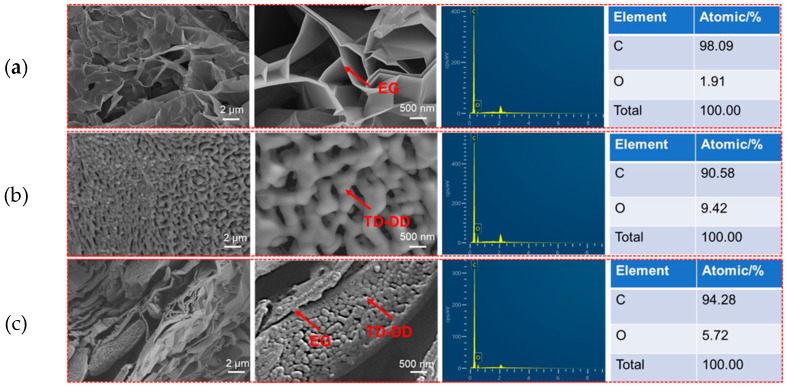
(**a**) SEM image and corresponding EDS analysis of EG; (**b**) SEM image and EDS analysis of TD-DD; (**c**) SEM image and EDS analysis of TD-DD/EG.

Figure 6 presents SEM and Energy-Dispersive X-ray Spectroscopy (EDS) results that confirm the successful integration and structural consolidation of TD-DD within the EG matrix. The characteristic porous network of pristine EG is shown in Figure 6a, where carbon is the dominant element (C: 98.09%, O: 1.91%), consistent with the intrinsic chemical composition of EG. In contrast, Figure 6c shows that the porous structure of EG has been effectively infiltrated by the TD-DD mixture. The corresponding hybrid microstructure, along with an intermediate oxygen content (5.72%), indicates a uniform distribution of TD-DD within the EG matrix. Compared to pure TD-DD, shown in Figure 6b, the reduced oxygen content in the composite reflects a dilution effect from EG and confirms the successful adsorption of TD-DD into the EG pores. Notably, the oxygen signal originates exclusively from TD-DD, confirming its presence within the composite. The EDS atomic percentage results (C: 94.28%, O: 5.72%) further reveal that TD-DD not only penetrates the porous architecture of EG but also forms a superficial coating layer, aligning well with the honeycomb-like cavity structure filled with TD-DD observed via SEM.

### 3.6. Thermal Property

**Figure 7:**
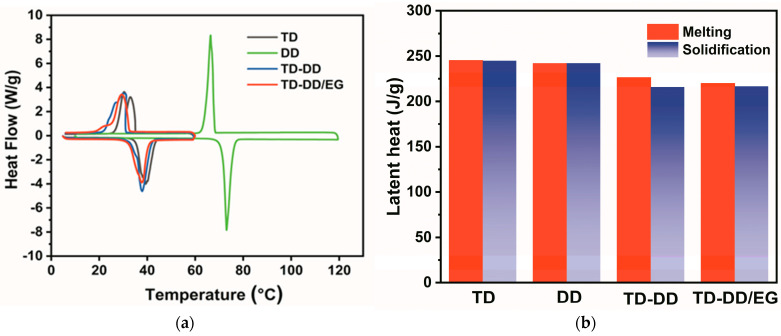
(**a**) DSC curves and (**b**) latent heat values of TD, DD, TD-DD, and the TD-DD/EG composite.

The phase change behavior of TD, DD, and their composite materials is systematically characterized by DSC, as shown in Figure 7. Both heating and cooling cycles are analyzed to comprehensively evaluate the thermal reversibility and practical applicability of the materials. In Figure 7a, pure TD and DD exhibit single endothermic peaks at 39.73 °C and 73.05 °C during melting, respectively, while the TD-DD eutectic mixture displays a sharp, single endothermic peak at 37.89 °C, confirming the successful formation of a homogeneous eutectic system. Correspondingly, during the solidification process, well-defined exothermic peaks are observed, further verifying the reversible phase transition behavior of the composites. The consistency between melting and solidification behavior underscores the thermal reliability of the material under cyclic operation. As shown in Figure 7b, further thermal analysis reveals that the TD-DD/EG composite melts at 37.77 °C, with a latent heat of 218.80 J/g, and solidifies at 29.38 °C, with a latent heat of 216.66 J/g. The suitable phase change temperature and considerable latent heat of the composite align well with the requirements for building energy efficiency applications.

**Figure 8:**
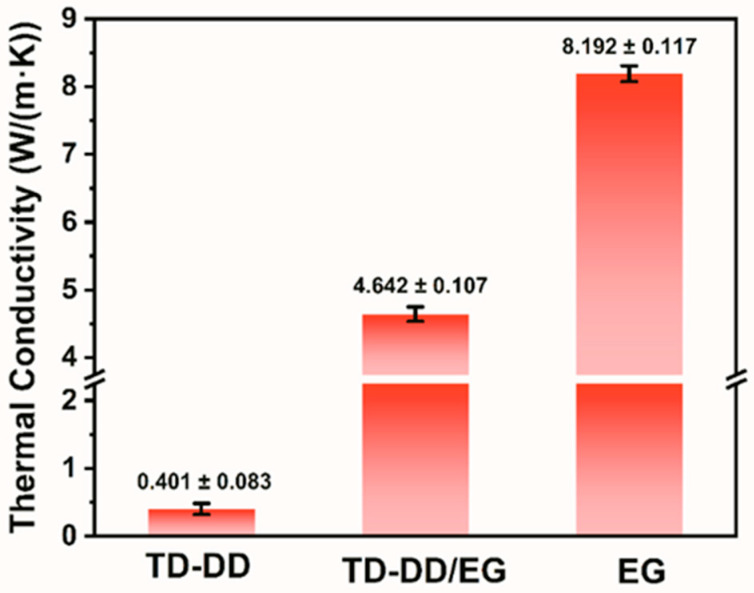
Thermal conductivity of EG, TD-DD, and TD-DD/EG.

The thermal conductivity of PCMs strongly influences their energy storage and release rates [32]. Thermal conductivity is measured at 25 °C using a transient plane source method. The apparatus, equipped with a kapton-coated sensor, records the heat transfer through two identical cylindrical sample disks (4 mm thick) under a constant contact pressure. As shown in Figure 8, the measured thermal conductivities are 8.192 W/(m·K), 0.401 W/(m·K), and 4.642 W/(m·K) for EG, TD-DD, and TD-DD/EG, respectively. The composite demonstrates a 12-times enhancement in thermal conductivity relative to pure TD-DD. The narrow standard deviations observed across replicates support the reproducibility of these measurements and confirm the consistent thermal conductivity improvement resulting from EG incorporation. This improvement arises from the notable intrinsic thermal conductivity and low density of EG, which together improve heat transfer and reduce the overall density of the composite [33]. The experimental results confirm that EG can effectively enhance the thermal conductivity of PCM based on TD-DD. As summarized in Table 2, compared with other materials reported in this field, it offers a suitable melting temperature of 37.77 °C and a melting enthalpy of 218.80 J/g, together with a thermal conductivity value 12 times that of the pure TD-DD system. These combined properties suggest potential applications of the material in sustainable building design.

### 3.7. Thermal Stability

**Figure 9:**
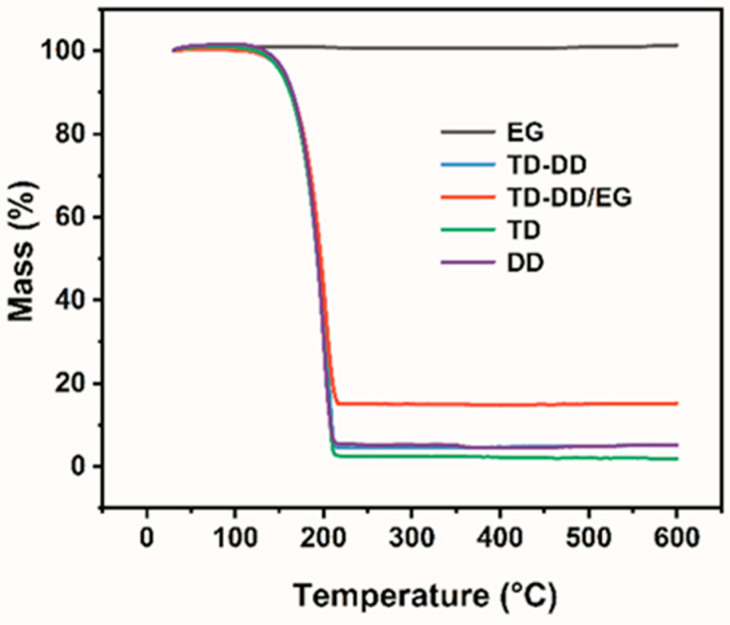
TG curves of TD, DD, EG, TD-DD, and TD-DD/EG.

As shown in Figure 9, EG shows a good thermal stability with a negligible mass loss at 600 °C. In contrast, TD, DD, and their eutectic blend TD-DD behave as typical organic materials, decomposing from around 130 °C and losing mass rapidly between 150 and 200 °C. At 600 °C, their residual masses are only 1.93%, 5.09%, and 5.30%, respectively. The TD-DD/EG composite retains a 15.22% residual mass at 600 °C, more than pure TD-DD (5.30%). The slightly higher value indicates that EG acts as a porous host that encapsulates TD-DD via a physical interaction, without chemical change. Moreover, the layered structure of EG acts as a barrier that partially confines the decomposition products of TD-DD, thereby increasing the char yield and verifying the enhanced thermal stability of the composite.

### 3.8. Cyclic Stability

**Figure 10:**
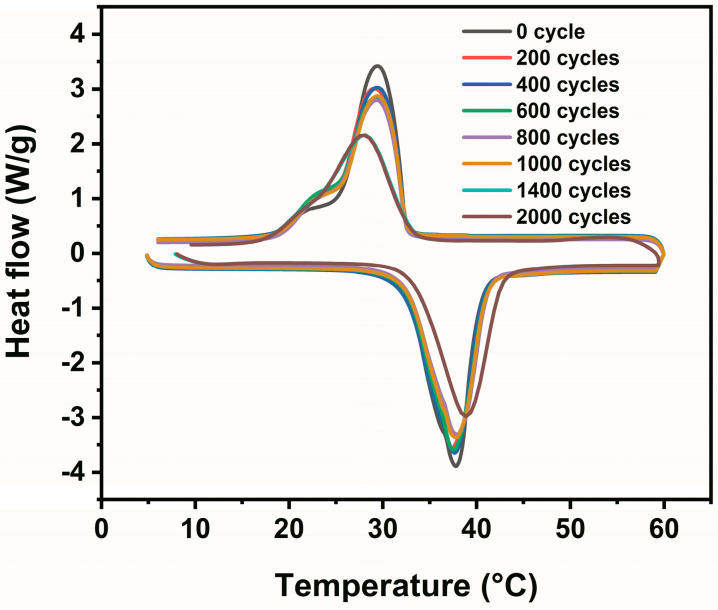
DSC curves characterizing the phase change behavior of the TD-DD/EG composite subjected to repeated thermal cycling.

The stability of the phase change temperature and latent heat determines whether a PCM can be used for long-term applications [38]. The thermal cycling stability of the TD-DD/EG composite PCM is evaluated through repeated phase transition cycles between 10 °C and 60 °C, as shown in Figure 10. The initial thermal behavior of the composite is characterized by distinct melting and solidification points at 37.77 °C and 29.38 °C, accompanied by latent heats of 218.80 J/g and 216.66 J/g, respectively. As summarized in Table 3, both the phase transition temperatures and enthalpies remain highly stable after 200, 400, 600, 800, and 1000 cycles. To further investigate its long-term reliability, testing is extended to 1400 and 2000 cycles. Even after 2000 cycles, the melting and solidification temperatures are well maintained at 38.05 °C and 28.88 °C, with melting and solidification enthalpies of 191.60 J/g and 186.73 J/g, respectively, representing a retention of over 87% of the initial melting enthalpy. The small shift in phase transition temperatures, coupled with the high enthalpy retention, demonstrates the good cycling stability of the composite. This stability is primarily attributed to the effective encapsulation of the TD-DD eutectic within the three-dimensional porous network of EG, which suppresses component migration and phase separation during phase transitions, thereby preserving the structural integrity and thermal performance of the material over extended cycling. When scaled to a typical building thermal energy management scenario, assuming one full phase change cycle per day, the 1000 and 2000 accelerated thermal cycles performed in this study correspond approximately to 2.7 and 5.5 years of actual service, respectively [39]. The high enthalpy retention and the gradually slowing degradation kinetics observed during these cycles indicate a promising potential for medium-term applications. It should be noted, however, that, while the accelerated testing provides strong evidence of the material’s intrinsic stability, its long-term performance under realistic and complex operating conditions (e.g., varying thermal loads, humidity, and mechanical stresses) requires further validation through application-oriented studies. In summary, the well-preserved thermal properties confirm the reliability of the composite PCM and support its potential for long-term use in TES systems.

## 4. Conclusions

A TD-DD/EG composite PCM was successfully fabricated through vacuum impregnation to overcome the inherent limitations of organic PCMs, including restricted temperature adaptability, molten-state leakage, and low thermal conductivity. Theoretical and experimental analyses confirm an optimal TD-DD eutectic mass ratio of 8:1, which exhibits a melting temperature of 37.89 °C (latent heat: 226 J/g) and a solidification temperature of 29.90 °C (latent heat: 216.00 J/g). The resulting TD-DD/EG composite retains a comparable phase change behavior, with melting and solidification temperatures of 37.77 °C (latent heat: 218.80 J/g) and 29.38 °C (latent heat: 216.66 J/g), respectively, and exhibits a low mass loss of 0.80% after 2 h at 60 °C. The composite achieves a thermal conductivity of 4.642 W/(m·K), approximately 12 times that of the pure TD-DD eutectic, along with a stable cycling performance: after 1000 thermal cycles, 93% of the original melting enthalpy (203.39 J/g) is retained, while the phase transition temperatures remain nearly unchanged. Upon extending the test to 2000 cycles, the material still retains about 87.60% of its initial latent heat (191.60 J/g), and the degradation rate shows a gradually slowing trend, indicating a good long-term thermal reliability. These findings demonstrate that the binary eutectic design combined with a porous EG matrix offers a viable strategy for developing high-performance composite PCMs. This work provides relevant data and a theoretical basis for potential applications in building energy efficiency and thermal storage systems, though further investigations under realistic conditions remain necessary to evaluate long-term durability. It is worth emphasizing that the material’s performance was evaluated under controlled laboratory settings, and its adaptability to complex practical scenarios (e.g., fluctuating humidity, intermittent mechanical stress) remains to be further validated. Furthermore, the vacuum impregnation method employed in this work may present obstacles to large-scale manufacturing, such as striking a balance between preparation efficiency and the uniform dispersion of the eutectic phase within bulk EG matrices.

## Figures and Tables

**Table 1:** Specific surface area and pore volume of EG and TD-DD/EG.

Samples	BET Surface Area (m^2^/g)			Pore Volume (cm^3^/g)		
	Micropore	Mesopore	Sum-Up	Micropore	Mesopore	Sum-Up
EG	4.01	37.21	41.22	0.0016	0.1100	0.1116
TD-DD/EG	negligible	0.12	0.12	negligible	0.0010	0.0010

**Table 2:** Thermal property comparison of some composites in the literature.

Samples	Temperature (°C)		Latent Heat (J/g)		Thermal Conductivity (W/(m·K))	References
	Melting	Solidification	Melting	Solidification		
TD-PA/EP-CF	33.60	29.70	138.30	137.50	1.081	[19]
CA-SA/EG	24.47	22.82	150.42	150.35	0.522	[34]
SA-ADA/EG	65.70	66.60	185.30	184.50	8.375	[35]
PA-SA/EG	53.89	54.37	166.27	160.13	2.510	[36]
CA-PA-SA/EG	28.93	16.32	137.38	141.51	3.677	[37]
TD	39.73	31.29	245.82	245.00	0.355	This work
DD	73.05	66.41	242.00	242.33	0.545	This work
TD-DD	37.89	29.90	226.00	216.00	0.401	This work
TD-DD/EG	37.77	29.38	218.80	216.66	4.642	This work

**Table 3:** Thermal properties of TD-DD/EG composite before and after thermal cycling.

Samples	Temperature (°C)		Latent Heat (J/g)	
	Melting	Solidification	Melting	Solidification
0 cycles	37.77	29.38	218.80	216.66
After 200 cycles	37.36	29.07	216.16	214.11
After 400 cycles	37.62	29.38	213.75	211.44
After 600 cycles	37.52	29.25	211.32	209.63
After 800 cycles	37.91	29.36	206.88	204.57
After 1000 cycles	37.78	29.35	203.39	202.19
After 1400 cycles	37.93	28.36	197.25	183.21
After 2000 cycles	38.05	28.88	191.60	186.73

## Data Availability

The original contributions presented in this study are included in the article. Further inquiries can be directed to the corresponding authors.

## Abbreviations

The following abbreviations are used in this manuscript:

TES	Thermal energy storage
PCM	Phase change material
TD	1-tetradecanol
DD	1, 10-decanediol
EG	Expanded graphite
OA	Octanoic acid
PA	Palmitic acid
CA	Citric acid
SA	Salicylic acid
ADA	Adipic acid
HDTMOS	Hexadecyltrimethoxysilane
EP-CF	Expanded perlite
DSC	Differential Scanning Calorimetry
TG	Thermogravimetric
SEM	Scanning Electron Microscopy
EDS	Energy-Dispersive X-ray Spectroscopy
BET	Brunauer–Emmett–Teller
BJH	Barrett–Joyner–Halenda
FT-IR	Fourier Transform Infrared
XRD	X-ray Diffractometry
